# Detecting Developmental Delay and Autism Through Machine Learning Models Using Home Videos of Bangladeshi Children: Development and Validation Study

**DOI:** 10.2196/13822

**Published:** 2019-04-24

**Authors:** Qandeel Tariq, Scott Lanyon Fleming, Jessey Nicole Schwartz, Kaitlyn Dunlap, Conor Corbin, Peter Washington, Haik Kalantarian, Naila Z Khan, Gary L Darmstadt, Dennis Paul Wall

**Affiliations:** 1 Division of Systems Medicine Department of Pediatrics Stanford University Palo Alto, CA United States; 2 Department of Biomedical Data Science Stanford University Palo Alto, CA United States; 3 Dhaka Shishu Children's Hospital Dhaka Bangladesh; 4 Division of Neonatal and Developmental Medicine Department of Pediatrics Stanford University Palo Alto, CA United States

**Keywords:** autism, autism spectrum disorder, machine learning, developmental delays, clinical resources, Bangladesh, Biomedical Data Science

## Abstract

**Background:**

Autism spectrum disorder (ASD) is currently diagnosed using qualitative methods that measure between 20-100 behaviors, can span multiple appointments with trained clinicians, and take several hours to complete. In our previous work, we demonstrated the efficacy of machine learning classifiers to accelerate the process by collecting home videos of US-based children, identifying a reduced subset of behavioral features that are scored by untrained raters using a machine learning classifier to determine children’s “risk scores” for autism. We achieved an accuracy of 92% (95% CI 88%-97%) on US videos using a classifier built on five features.

**Objective:**

Using videos of Bangladeshi children collected from Dhaka Shishu Children’s Hospital, we aim to scale our pipeline to another culture and other developmental delays, including speech and language conditions.

**Methods:**

Although our previously published and validated pipeline and set of classifiers perform reasonably well on Bangladeshi videos (75% accuracy, 95% CI 71%-78%), this work improves on that accuracy through the development and application of a powerful new technique for adaptive aggregation of crowdsourced labels. We enhance both the utility and performance of our model by building two classification layers: The first layer distinguishes between typical and atypical behavior, and the second layer distinguishes between ASD and non-ASD. In each of the layers, we use a unique rater weighting scheme to aggregate classification scores from different raters based on their expertise. We also determine Shapley values for the most important features in the classifier to understand how the classifiers’ process aligns with clinical intuition.

**Results:**

Using these techniques, we achieved an accuracy (area under the curve [AUC]) of 76% (SD 3%) and sensitivity of 76% (SD 4%) for identifying atypical children from among developmentally delayed children, and an accuracy (AUC) of 85% (SD 5%) and sensitivity of 76% (SD 6%) for identifying children with ASD from those predicted to have other developmental delays.

**Conclusions:**

These results show promise for using a mobile video-based and machine learning–directed approach for early and remote detection of autism in Bangladeshi children. This strategy could provide important resources for developmental health in developing countries with few clinical resources for diagnosis, helping children get access to care at an early age. Future research aimed at extending the application of this approach to identify a range of other conditions and determine the population-level burden of developmental disabilities and impairments will be of high value.

## Introduction

Autism spectrum disorder (ASD) is a heterogeneous developmental disorder that includes deficits in social communication, repetitive behaviors, and restrictive interests, all of which lead to significant social and occupational impairments throughout the lifespan. Autism is one of the fastest growing developmental disorders in the United States [1], affecting 1 in 59 children [2]. Although the global autism prevalence is largely unknown, the prevalence is estimated to be between 0.15% and 0.8% among children in developing countries such as Bangladesh, with a higher prevalence in urban centers (eg, 3% in Dhaka) [3]. These numbers only represent a fraction of the actual cases, as most cases in semiurban and rural areas go unnoticed due to a dearth of resources. The disparity between urban and rural prevalence may reflect poorly understood risk factors or clinical resources in high-income areas along with higher awareness among urban parents about developmental delays [4]. More accessible and wide-scale screening is needed to accurately estimate ASD prevalence in remote parts of Bangladesh and other countries.

The current models for diagnosing autism in Bangladesh, as in the United States, are often administered by trained clinical professionals using standard assessments [5]. Empirically validated diagnostic tools like the Autism Diagnostic Observation Schedule (ADOS) [6] and Autism Diagnostic Interview (ADI-R) [7] are not always used in different countries, particularly in developing countries, as these tools are expensive, require trained clinicians to administer, and may be limited by available translations and cultural adaptations [4]. For countries with limited ASD resources like Bangladesh, obtaining a diagnosis, which is essential for receiving an intervention and improving outcomes, is difficult. There is a pressing need to further develop open-source tools that do not require extensive training and professional certification and have high cross-cultural validity for autism screening globally [4]. Previous work has shown the feasibility and efficacy of assessing developmental delay using rapid assessment tools delivered by professionals with limited clinical expertise in the home [5]. There is potential to extend the reach of assessment tools and decrease health care disparity, especially in developing and rural countries, by using machine learning and mobile technologies.

In our previous works, we have developed tools for rapid mobile detection of ASD in short home videos of US children by using supervised machine learning approaches to identify minimal sets of behaviors that align with clinical diagnoses of ASD [8-15]. Features extracted in our minimally viable classifiers are accurately labeled by nonexpert raters (ie, noncertified clinical practitioners) in a short period of time (eg, <6 minutes). These labeled features can then be fed into our machine learning classifiers to determine the child’s autism risk. Tariq et al [14] used a dataset consisting of 162 videos (116 ASD, 46 neurotypical development [TD]) of US children to validate these classifiers. The top-performing classifier exhibited an accuracy of 92% (95% CI 88%-97%).

Additionally, an independent validation set consisting of 66 videos (33 ASD, 33 TD) was labeled by a separate set of video raters in order to validate the results. The top-performing classifier maintained similar results, achieving an overall accuracy of 89% (95% CI 81%-95%).

The current study aimed to show generalizability of video-based machine learning procedures for ASD detection that have established validity among US-based children [14] in Bangladesh. Specifically, our study aimed to determine the performance and accuracy of this same video machine learning procedures on videos of Bangladeshi children under the age of 4 years. This sample was drawn from a population diagnosed with ASD and another population with other speech and language conditions (SLCs), but not ASD. Additionally, we compared the features that are most important for accurate classification of children from Bangladesh and created several machine learning models that can be generalized to different cultures.

## Methods

### Data Collection

The study received ethical clearance under Dr Naila Khan from the Bangladesh Institute of Child Health, Dhaka Shishu Children’s Hospital (DSH) and the Stanford University Institutional Review Board. We aimed to recruit 150 children for this study: 50 with ASD, 50 with an SLC, and 50 with neurotypical development (TD). All participants were recruited after they provided consent (in Bengali language) for participation at the DSH, and their children were screened for the presence of ASD or SLC. Participants were enrolled if they were parents above 18 years of age, had a child between the ages of 18 months and 4 years, could attend an appointment at the DSH to complete the study procedures, and were willing to submit a brief video of their child to the study team. Enrolled families provided demographic information (see Table 1 in the Results section).

Brief videos (2-5 minutes) were recorded during evaluation of the children who presented to the Child Development Center of the Bangladesh Institute of Child Health with neurodevelopmental concerns. We administered the Modified Checklist for Autism in Toddlers (Bangla version [16]) to all children to identify the presence of ASD, and all children underwent additional clinical evaluations by a developmental psychologist and a child health physician in order diagnose ASD, SLC, or TD, as described previously [5]. We also administered the ADOS for 28 of the 50 children identified with ASD; ADOS could not be completed in the remaining 22 children diagnosed with ASD because their families were unable to commit to the time required to complete the assessment, a common problem for families in low-resource areas [4].

Acquired videos and supporting demographic measures were securely sent from DSH to Stanford University. Videos were assessed for quality by trained clinical researchers at Stanford University. Criteria included video, sound, and image quality in addition to video length and content (ie, ensuring that the video was long enough to answer necessary questions, that the child was present in the video, etc). Furthermore, videos were assessed to meet the following criteria: (1) it captured the child’s face and hands, (2) it involved social interaction or attempts of social interaction, and (3) it involved an interaction between the child and a toy/object.

### Video Raters

Nine non-Bengali speaking US-based raters with no clinical training used a secure, HIPAA (Health Insurance Portability and Accountability Act)-compliant online website to watch the videos and answer a set of 31 multiple-choice questions corresponding to the behavioral features of autism [14]. Each rater completed a 1-hour training session with a senior analyst before scoring the videos. Senior analysts conducted rater quality checks by comparing a subset of 10 video scores to “gold standard” scores. These “gold standard” scores were agreed upon by two clinical research coordinators who each had several years of experience with children with autism.

### Source Classifiers Trained on Clinical Data for Reduce-to-Practice Testing

We assembled eight published machine learning classifiers to test their viability for use in the rapid mobile detection of autism through the use of short home videos of US children [14]. For all eight models, the source of training and validation data was item-level medical records of US children, which contained either the ADOS or ADI-R outcome data on all participants. The ADOS has several modules containing approximately 30 features that correspond to the developmental level of the individual under assessment. These features are assessed based on how a child interacts with a clinical practitioner administering the exam. The ADI-R is a parent-directed interview that includes >90 elements asked of the parent, with multiple choices for answers. Each model was trained on item-level outcomes from the administration of either the ADOS or ADI-R and optimized for accuracy, sparsity of features, and interpretability in previous publications [8-15]. All these classifiers have been validated with US home videos (total: n=162, ASD: n=116, non-ASD: n=46) [14]. The top three performing classifiers in this dataset were chosen for validation of the videos collected from DSH in Bangladesh to test the accuracies of these models across cultures.

### Stacked Classifiers With Rater-Adaptive Weighting

In an effort to improve the results on the Bangladeshi dataset after attempting to validate previously built classifiers on these data, we constructed new classifiers while controlling for potential noise resulting from inaccurate ratings and constructed separate layers for each step of the classification for a streamlined approach. Our dataset contained three classes—TD, ASD, and SLC—assigned by screening via clinical evaluation at the DSH [5]. By implementing a layered approach to classification—first distinguishing general developmental delays (including ASD and SLC) from TD and then distinguishing ASD from SLCs—we were able to broaden the detection capabilities to more generally classify the presence of other developmental delays in addition to ASD specifically.

#### Rater Weighting

Given the raters’ lack of formal clinical training, we hypothesized that some raters might be more adept at identifying certain risk factors in some videos than others. Regardless of whether these interrater differences in identification accuracy for certain subsets of behaviors arise naturally or by chance, we hypothesized that this heterogeneous rater performance could be leveraged to yield increased model performance. For example, if one rater is especially capable of labeling a child’s level of eye contact and another rater does a poor job of rating eye contact but excels at rating language ability, then a model trained on each individual rater’s labels alone might perform poorly; however, an ensemble that considers the outputs of both rater’s models could perform substantially better. Achieving this improved performance is the focus of our proposed novel rater-adaptive weighting scheme.

For each of the three raters in the dataset, we trained a Random Forest classifier to predict a child’s class label (TD, SLC, or ASD) based on the rater’s annotations of that child’s behavior in a given video. The Random Forest classifier adapts to each rater’s expertise and labeling patterns; a basic analysis revealed that each rater had a different feature set that they rated well. In addition to (and, in part, because of) interrater differences in the labeling ability, each rater’s model had varying levels of accuracy. We wanted the ensemble to weigh the predictions from the most accurate rater models more heavily. Therefore, we first trained and calculated the accuracy of each rater’s model relative to a majority vote baseline and then used that difference to up- or downweigh that rater’s vote relative to the other raters’ votes.

Specifically, we let z_j_ represent the difference in accuracy of rater *j* ’s model relative to the majority vote baseline. Then, after calculating z_j_ for each rater j=1, 2...K, we pass these values into the softmax function to generate rater-specific weights:


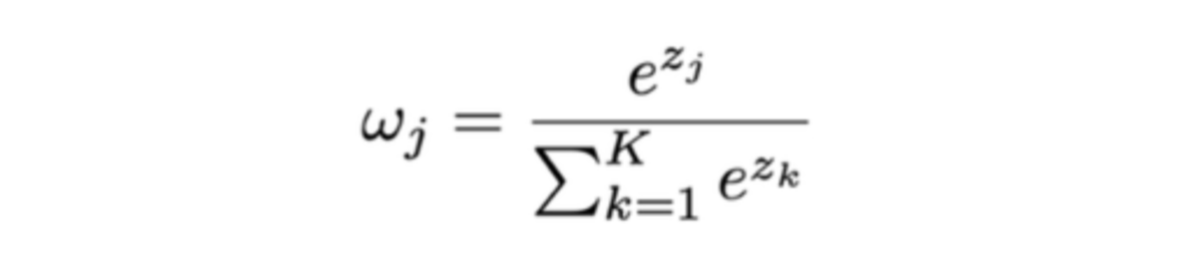


This ensures that all the raters’ weights collectively sum up to 1, so that the ensemble prediction will be a linear combination of each rater’s predictions. Using these weights, the final ensemble prediction for child *i* is calculated by multiplying each rater model’s predicted probability for the target class (eg, atypical development or ASD) by the corresponding rater-specific weight and adding the weighted raters’ predicted probabilities together. More specific details can be found in Multimedia Appendix 1.

#### Stacking Classifiers to Distinguish Between Typical/Atypical Development and Autism Spectrum Disorder/Speech and Language Conditions

In order to reflect the differences in both the conceptualization and use cases of predicting (1) TD vs atypical development and (2) ASD from other developmental delays, we decided to create a stacked approach to classification. In the first layer, we built classifiers to distinguish between TD and atypical development (ASD/other SLCs). The cases classified as atypical from the first layer were then used as input for the second layer to distinguish between ASD and other SLCs.

We wanted to optimize the model for sensitivity in the first layer to ensure no atypical case was misclassified. In the second layer, we wanted to optimize for both sensitivity and specificity, so that children with ASD would be effectively distinguished from children with other development delays. After training these classifiers for each rater, we tested them on the held-out test set and aggregated rater scores using the rater weights calculated in the previous step. For each of these layers, we used a three-fold cross-validation approach to select the training and test sets randomly in order to ensure that the accuracy reported is stable across different splits.

### Feature Importance

To determine the impact of each video’s annotations on the classifier’s predicted label for that video, we used a recently developed method for efficiently calculating approximate Shapley values [17]. Shapley values are traditionally used in coalitional game theory to determine how to optimally distribute gains earned from cooperative effort. The same idea can be extended to machine learning in order to rank features for nonlinear models such as Random Forests. In the machine learning adaptation of Shapley values, feature values “cooperate” to impact a machine learning model’s output, which in this case is the predicted probability of a child’s video being classified as TD, ASD, or SLC. For each video, Shapley values capture both the magnitude of importance for each feature value as well as the direction in which the feature value “pushes” the final predicted class probability. More precisely, if we let Φ_k_(F_j_*, x^(i)^) be the impact (Shapley value) of the *k* th feature for video *i* with feature vector x^(i)^ on the output of model F_j_*, then the Shapley value formulation guarantees that


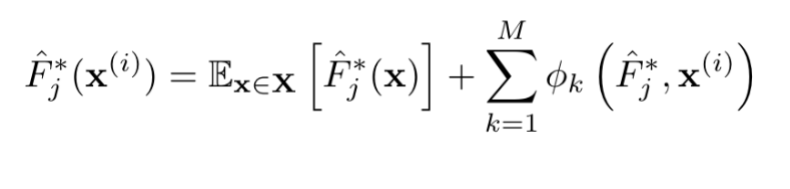


In other words, any video’s final predicted class probability is the average predicted class probability of the dataset plus all the Shapley values associated with each element of that video’s input vector. This property, called local accuracy, indicates that the feature importance can be easily measured and compared. Additionally, because each video, feature, and model triple is associated with a single scalar-valued feature importance, we can understand how each annotation for each child’s video affected his/her predicted probability of TD/ASD/SLC at an individual level and estimate a feature’s overall importance to the model by summing up the absolute values of that feature’s Shapley values over all videos. The features with the highest sum of absolute Shapley values are considered the most important to the model. Finally, given the way in which we ensembled individual raters’ models, we can extract Shapley values for the multirater ensemble by employing the same weights. Specifically, we can employ the following equation:


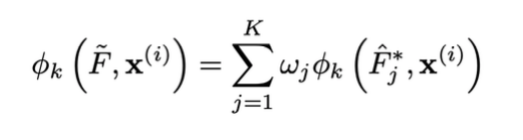


To test whether our classifier’s decisions align with clinical intuition, we calculated Shapley values for the 159 videos for the second layer of the classifier when distinguishing ASD from non-ASD.

### Comparing Bangladeshi and US Results

In order to determine the generalizability of one dataset’s characteristics to the other, we trained logistic regression classifiers with elastic net regularization for the Bangladeshi data and US data to predict ASD from the non-autism class. We trained the model on the Bangladeshi data and tested the model on the US data and vice versa. For both classifiers, we randomly split the dataset into training and testing, reserving 20% for the latter while using cross-validation on the training set to tune hyperparameters associated with elastic net regularization. Note that while traditional logistic regression seeks to find a set of model coefficients, β, that minimizes the logarithmic loss (we will denote this loss as 
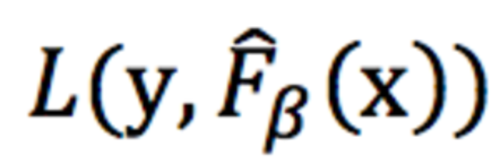
 where 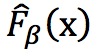
represents the model’s predictions when the model is parameterized by β), logistic regression with elastic net regularization seeks to minimize the logarithmic loss plus a regularization term:


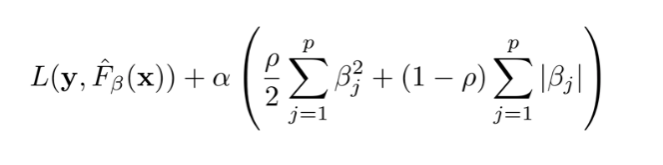


Here, the first sum corresponds to an L2-loss, the second sum corresponds to an L1-loss, *ρ* is a hyperparameter governing the balance between the two losses, and *α* is the second hyperparameter determining the overall strength of regularization. Incorporating this regularization into the logistic regression loss yields several benefits, including more parsimonious and interpretable models and better predictive performance, especially when two or more of the predictor variables are correlated [18]. We used cross-validation for model hyperparameter tuning by performing a grid search with different values of *α* (varying penalty weights) and *ρ* (the mixing parameter determining how much weight to apply to L1 versus L2 penalties) [19,20-21]. Based on the resulting area under the curve (AUC) and accuracy from each combination, we selected the top-performing pair of hyperparameters. Using this pair, we trained the model using logistic regression and balanced class weights to adjust weights that were inversely proportional to class frequencies in the input data, which helps account for class imbalance. After determining the top-ranked features based on the trained model and the resulting coefficients, we validated the model on the reserved test set. The behavioral features that were selected most often during the hyperparameter tuning phase across different cross-folds were compared between US and Bangladeshi models to determine which features have a greater significance and whether they align between the two models.

### Software

Analyses were performed in Python 3.6.7; we used pandas 0.23.4 to prepare the data for analysis [20]. The classification models described were trained and evaluated using the scikit-learn 0.20.0 package [21]. Hyperparameters for each rater model were tuned using the hyperopt 0.1.1 package [22]. Shapley value estimates were calculated using the shap 0.24.0 package [23]. Plots were generated using matplotlib 3.0.1 [24].

## Results

### Data Collection

We collected 159 videos in total: 55 videos were of children with ASD, 50 were of children with SLC, and 54 were of children with TD. The parent-submitted home videos were an average of 3 minutes 11 seconds long (SD 1 minute 57 seconds). Of the 159 videos submitted, all were manually inspected and found to be of good, scorable quality in terms of length, resolution, and content. Demographic data were missing for 9 subjects, who were excluded from analysis; all other data were complete. Video rating staff were able to rate all videos. Table 1 outlines the diagnosis and demographic breakdown for 150 of the 159 videos included in the dataset.

### Results of Source Classifiers Trained on Clinical Data for Reduce-to-Practice Testing

**Figure 1:**
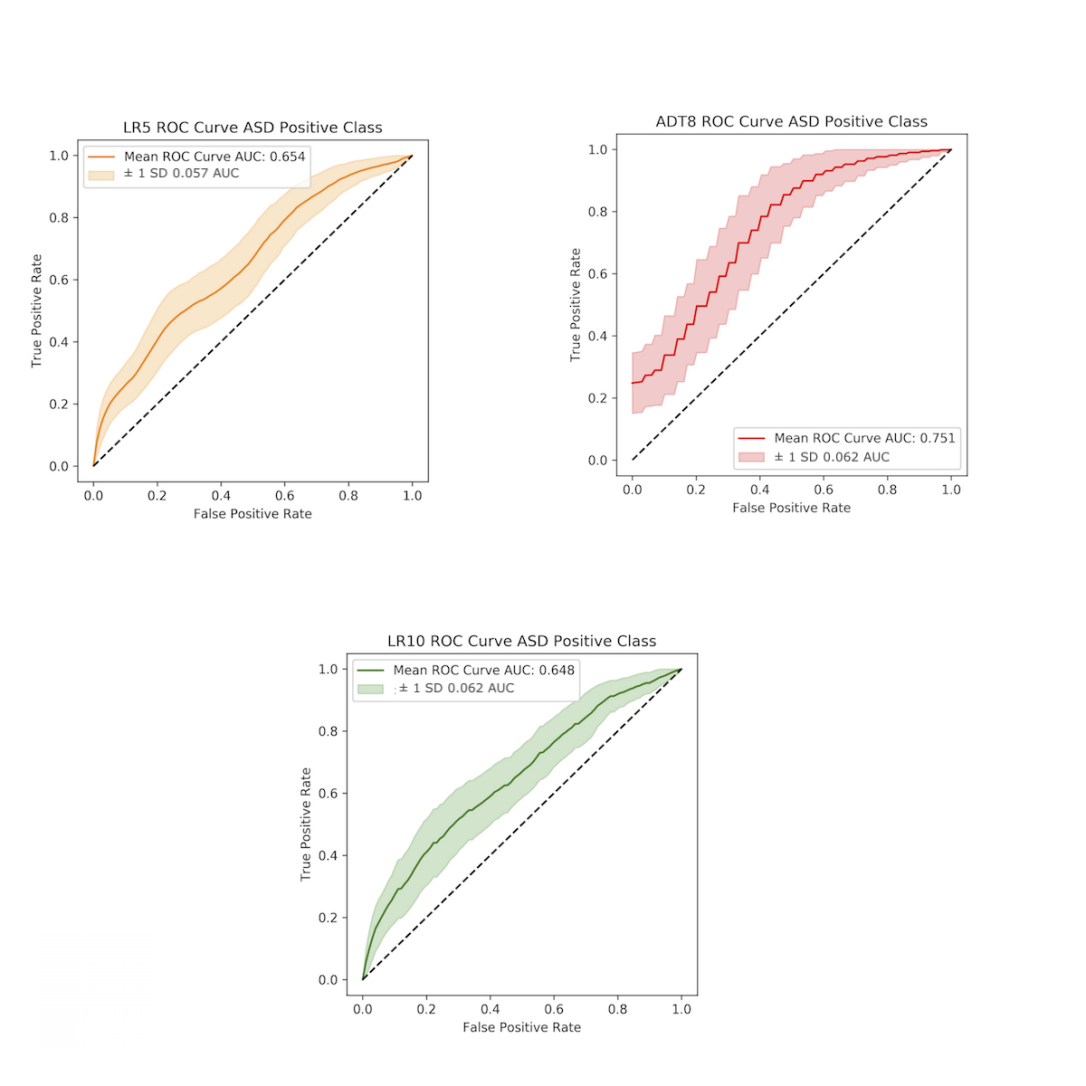
Results from the top performing classifiers trained on US clinical score sheet data and tested on Bangladeshi data with an objective to distinguish between ASD and non-ASD. ROC: receiver operating characteristic; AUC: area under the curve; ASD: autism spectrum disorder.

We first sought to distinguish AD from non-ASD cases. Our top performing classifiers from our previous analysis of the videos from 162 US children [14] were validated on the Bangladeshi dataset. We tested across different train-test splits and achieved a maximum AUC of 0.75 (SD 0.06; Figure 1). In order to improve classifier performance, we next shifted to the development of stacked classifiers.

### Results From Stacked Classifiers With Rater-Adaptive Weightings

Since we used a three-fold cross-validation approach, we trained and tested the models for each of the raters across three different splits. The training set consisted of 114 randomly selected videos, and the average demographic information for the three splits for the training set was as follows: average age, 2 years 7 months (SD 7 months); proportion of males, 64%; proportion of children with TD, 34%; proportion of children with SLC, 31%; and proportion of children with ASD, 35%. The demographic information for the test set for layer 1 (distinguishing TD from ASD/SLC) and layer 2 (distinguishing ASD from SLC) can be found in Table 2.

**Table 1:** Participant demographics collected from Dhaka Shishu Hospital, Bangladesh.

Demographic			Full cohort (N=150)	ASD^a^ cohort (N=50)	TD^b^ cohort (N=50)	SLC^c^ cohort (N=50)
Age (years), mean (SD)			2.55 (0.62)	2.51 (0.70)	2.40 (0.59)	2.73 (0.51)
Gender (male), n (%)			90 (60)	36 (72)	23 (62)	31 (46)
Preterm (ie, <37 weeks), n (%)			11 (0.7)	5 (10)	0 (0)	6 (12)
**Family income in taka^d^, n (%)**						
	1,000-10,000		16 (10.7)	0 (0)	16 (32)	0 (0)
	>10,000-30,000		33 (22)	2 (4)	21 (42)	10 (20)
	>30,000		101 (67.3)	48 (96)	13 (26)	40 (80)
**Residence, n (%)**						
	Urban		139 (92.7)	50 (100)	50 (100)	39 (78)
	Semiurban		8 (5.3)	0 (0)	0 (0)	8 (16)
	Rural		3 (2)	0 (0)	0 (0)	3 (6)
**Religion, n (%)**						
	Muslim		141 (94)	44 (88)	49 (98)	48 (96)
	Hindu		6 (4)	4 (8)	0 (0)	2 (4)
	Christian		1 (0.01)	1 (2)	0 (0)	0 (0)
	Buddhist		2 (0.01)	1 (2)	1 (2)	0 (0)
**Stunted growth, n (%)**						
	Missing stunting information		60 (40)	4 (8)	50 (100)	6 (12)
	No stunting		49 (32.7)	30 (60)	0 (0)	19 (48)
	Stunting		41 (27.3)	16 (32)	0 (0)	25 (50)
**Clinical evaluations, mean (SD)**						
	**MCHAT^e^ total score**			**13.5 (3.04)**	**2 (0)**	**0.08 (0.57)**
	**ADOS^f,g^ score**					
		Social affect	N/A^h^	11.57 (5.30)	N/A	N/A
		Restricted and repetitive behavior	N/A	3.46 (3.29)	N/A	N/A
		Composite	N/A	5.14 (2.08)	N/A	N/A
	**SLC diagnosis**					
		Receptive language delay	N/A	N/A	N/A	2 (4)
		Expressive language delay	N/A	N/A	N/A	5 (10)
		Both receptive and expressive language delay	N/A	N/A	N/A	37 (74)
		Receptive and expressive language disorder	N/A	N/A	N/A	6 (12)

^a^ASD: autism spectrum disorder.

^b^TD: neurotypical development.

^c^SLC: speech and language condition.

^d^1 US $=84 taka.

^e^MCHAT: Modified Checklist for Autism in Toddlers

^f^ADOS: Autism Diagnostic Observation Schedule.

^g^ADOS was only performed on a subset of 28 children with ASD.

^h^N/A: not available.

**Table 2:** Average demographic information of the test set calculated by testing the model on 45 videos for both layers.

Demographic	Layer 1 (distinguishing TD^a^ from ASD^b^/SLC^c^)	Layer 2 (distinguishing ASD from SLC)
Age (years), average (SD)	2 years 7 months (5 months)	2 years 6 months (3 months)
Proportion of males, mean %	62	70
Proportion of TD children, mean %	33	22
Proportion of children with ASD, mean %	33	44
Proportion of children with SLC, mean %	33	34

^a^TD: neurotypical development.

^b^ASD: autism spectrum disorder.

^c^SLC: speech and language condition.

**Figure 2:**
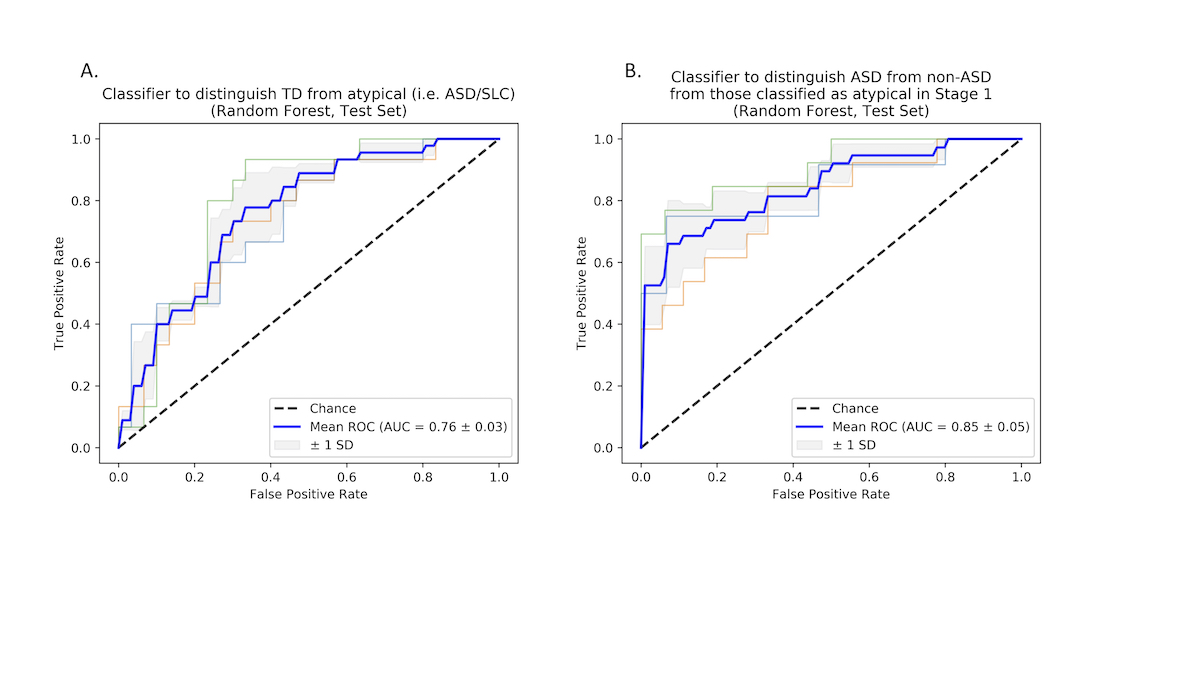
(A) ROC curve for layer 1 (distinguishing between children with TD and children with ASD or SLC). (B) ROC curve for layer 2 (distinguishing between ASD and SLC). ASD: autism spectrum disorder; AUC: area under the curve; SLC: speech and language condition; TD: neurotypical development; ROC: receiver operating characteristic.

**Table 3:** Results from classifiers to distinguish among autism spectrum disorder, speech and language conditions, and neurotypical development. The results distinguish layer 1 (distinguishing neurotypical development from atypical conditions [autism spectrum disorder/speech and language conditions]) and layer 2 (distinguishing autism spectrum disorder from other delays [speech and language conditions]) from those classified as atypical in layer 1.

Classifier Layer	Sensitivity, % (SD)	Specificity, % (SD)	Unweighted average recall, % (SD)	Area under the curve, % (SD)	Accuracy, % (SD%)
Layer 1^a^	76 (SD 4)	58 (SD 3)	67 (SD 1)	76 (SD 3)	70 (SD 2)
Layer 2^b^	76 (SD 6)	77 (SD 24)	77 (SD 9)	85 (SD 5)	76 (SD 11)

^a^Distinguishing neurotypical development from autism spectrum disorder/speech and language conditions.

^b^Distinguishing autism spectrum disorder from other developmental delays (speech and language conditions).

Layer 1 of the stacked classifier, which sought to distinguish between children with TD from children with atypical development, achieved 76% (SD 4%) sensitivity and 58% (SD 3%) specificity with an AUC of 76% (SD 3%) and an accuracy of 70% (SD 2%; Figure 2 A). For layer 2, which distinguished ASD from other SLCs, the classifier performed with 76% (SD 6%) sensitivity, 77% (SD 24%) specificity with an AUC of 85% (SD 5%) and accuracy of 76% (SD 11%; Figure 2 B; Table 3).

### Feature Importance

**Figure 3:**
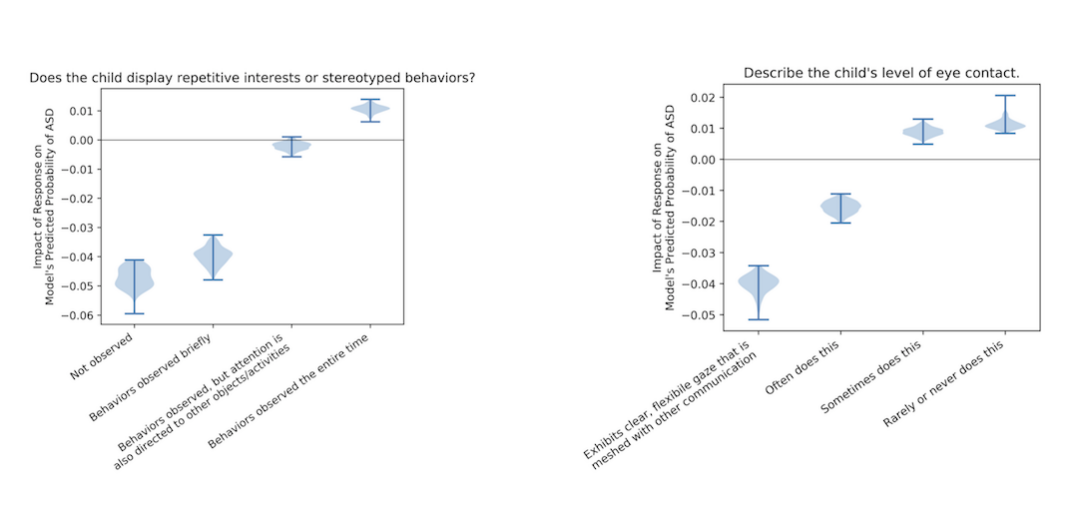
Shapley value distributions for two of the most important features in the rater-adaptive ensemble model. These features measure the child’s stereotyped behaviors/repetitive interests and eye contact. They demonstrate that clinical intuition and the inner workings of our classifier align closely. ASD: autism spectrum disorder.

The most important features in our rater-adaptive ensemble for predicting ASD, as measured by the Shapley value, align with clinical intuition. Figure 3 shows the distribution of Shapley values across all participants for two of the features that were among the most important (as measured by mean absolute Shapley value) to our ensemble model’s predictions. For example, for the feature corresponding to the child’s level of eye contact, the value “rarely or never does this” contributes strongly to a classification of ASD and “exhibits clear, flexible gaze that is meshed with other communication” contributes the most to a non-ASD classification. Another feature that aligns with clinical intuition measures the child’s repetitive interests and stereotyped behaviors—the feature value “behaviors observed the entire time,” contributes strongly to the positive class (ASD), whereas “not observed” contributes strongly to the negative class (non-ASD; Figure 3).

### Comparison of Bangladeshi and US Results

**Figure 4:**
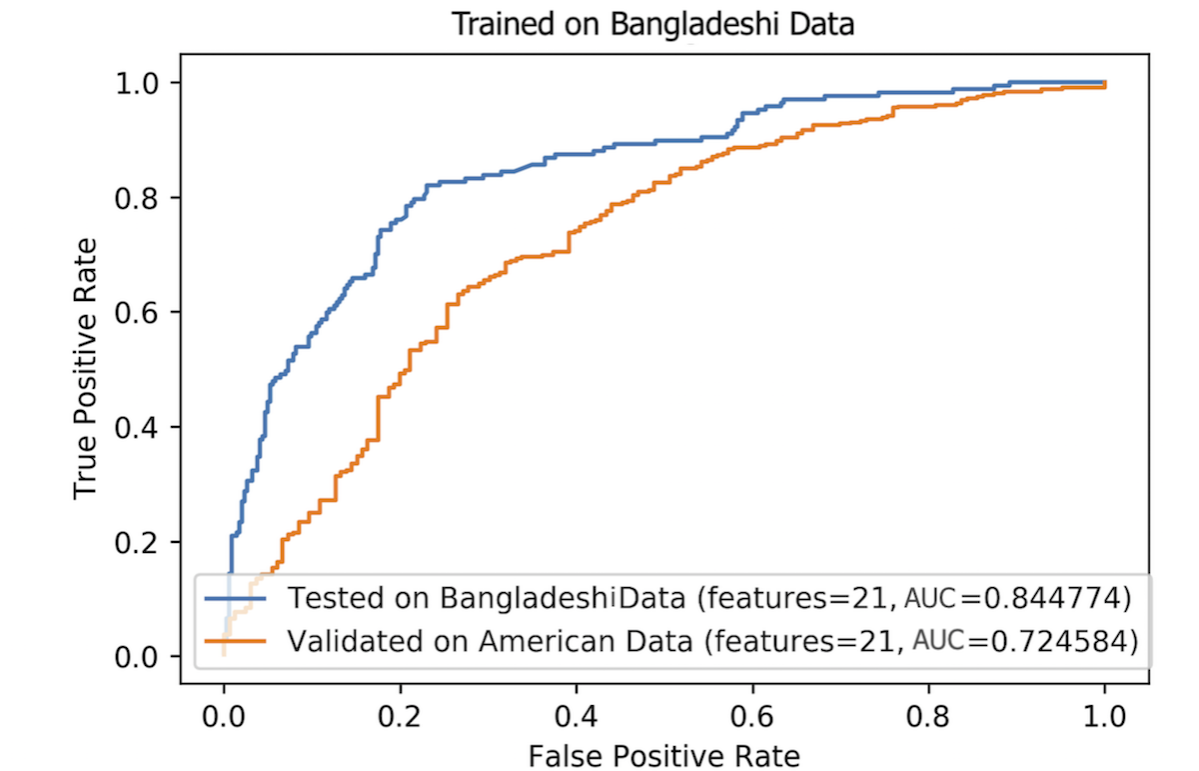
Logistic regression (Elastic Net penalty) classifier, trained on Bangladeshi data and tested on US data as well as a held-out test set of the Bangladeshi data. AUC: area under the curve.

For the classifier trained on the Bangladeshi data, the performance on the held-out test set (20% of Bangladeshi data) was 84.4% and its performance when validated on US data was 72.5% (Figure 4).

**Figure 5:**
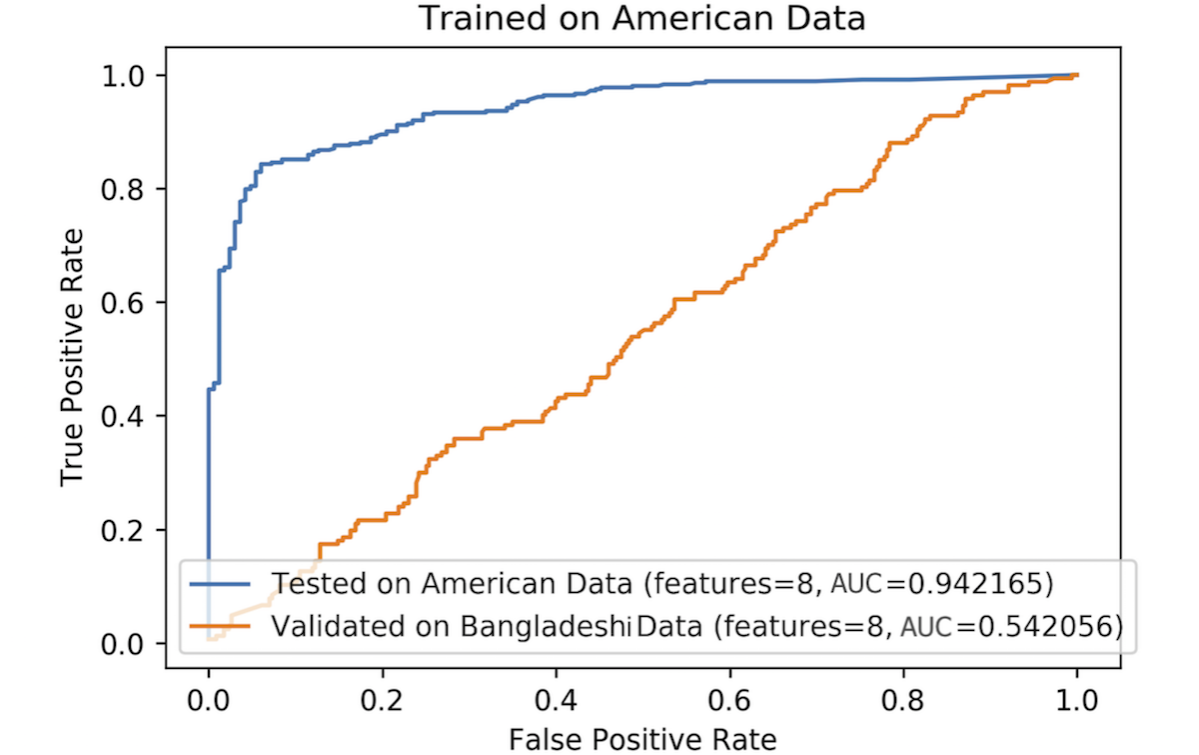
Logistic regression (Elastic Net penalty) classifier, trained on US data and tested on Bangladeshi data as well as a held-out test set of the US data.

We trained a similar classifier on our dataset of 162 US videos and validated it on the Bangladeshi data (Figure 5). The classifier performed with a 94.2% accuracy when tested on the held-out test set from US videos. The classifier’s accuracy dropped significantly when validated on the Bangladeshi data, reaching around 54%.

**Figure 6:**
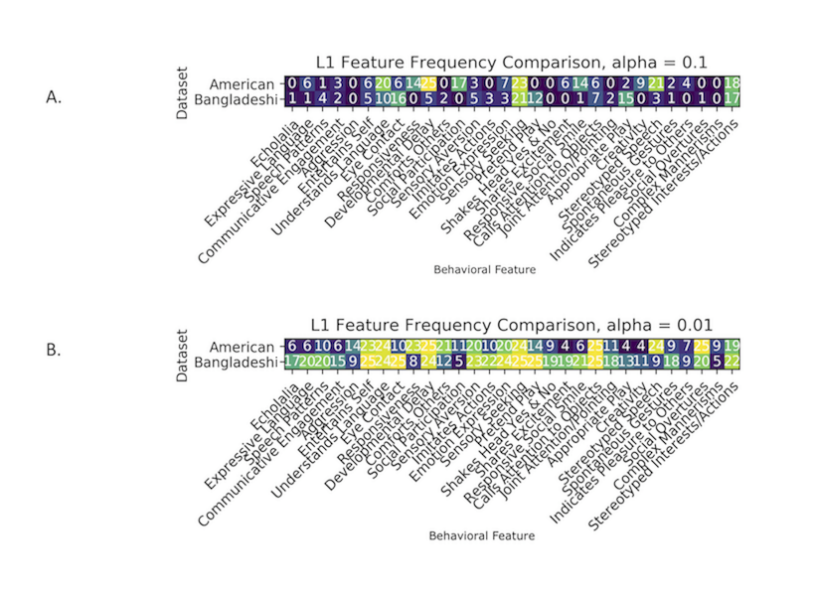
Feature selection analysis. Numbers within the cells indicate the frequency of selection. (A) Feature frequency comparison during cross-fold validation with alpha value 0.1 between Bangladeshi data and US data. (B) Feature frequency comparison during cross-fold validation with alpha value 0.01 between Bangladeshi data and US data.

While performing hyperparameter tuning on these classifiers, we conducted further analysis to determine which of the behavioral features were selected most often for each cross-fold of US videos and Bangladeshi videos in order to draw a comparison. It is apparent from Figure 6 A and 6B that the features being selected are quite similar between the two datasets, with some minor differences. The features *understands language, sensory seeking, calls attention to objects*, and *stereotyped interests and actions* are highly ranked by models trained on either of the datasets. *Responsiveness, developmental delay, social participation,* and *stereotyped speech* are selected more often for US data and less so for Bangladeshi data. The opposite is true for *eye contact*.

## Discussion

### Principal Results

We were able to demonstrate the potential to use video-based machine learning methods to detect developmental delay and autism in a collection of videos of Bangladeshi children at risk for autism. Despite language, cultural, and geographic barriers, this outcome shows promise for remote autism detection in a developing country. More testing and refinement will be needed, but, in general, there is potential for the method to be made virtual to run entirely on mobile devices and therefore potential to increase the capacity to detect and provide more immediate diagnostics to children in need of therapeutic interventions.

An important result of our work is that we were able to gather 159 videos from Bangladeshi parents collected via mobile phone through our collaboration with DSH. This suggests feasibility of expanding this study to a larger sample size across Bangladesh and other low-resource settings and the ability to rely on the use of mobile phones in developing countries like Bangladesh, where 95% of the population are mobile phone subscribers [25]. Additionally, we found that clinically untrained, US-based, non-Bengali speaking raters were able to score videos of Bangladeshi children with limited training, suggesting that speaking the native language may not be necessary for scoring videos. This finding also demonstrates the validity and potential of this mobile tool to be deployed across cultures and languages.

A useful and novel contribution of our work was our method for ensembling predictions from models trained on and adapted to each individual rater. This method demonstrates several advantageous properties. First, because each classification model was trained to map an individual rater’s annotation patterns to a predicted class label, these rater-adaptive models can capitalize on features reflecting a rater’s strengths while ignoring features on which the rater shows weaker performance. Furthermore, the fact that raters’ models are trained independently from one another means that, in a distributed setting where there is a large corpora of videos such that each rater annotates only a small subset of them, our method can make predictions on each video by applying and ensembling the models from each rater without any need for additional imputation. By weighting each rater’s model according to its accuracy on a rater-specific held-out validation set, the overall ensemble can lean more heavily on those raters whose models consistently demonstrate the best classification performance. Finally, because the final ensemble’s prediction is a linear combination of all of the rater’s models and we are able to calculate Shapley values for every feature in each of these models, it follows that we can use the same weights from the ensemble of rater-specific predictions to generate ensemble-level Shapley values as well. Thus, if a child’s video is distributed to several different raters and those raters’ annotations are fed into the ensemble model, one can interpret how each of the child’s behavioral annotations contributed to both the final ensemble classification label and each rater’s predicted label individually.

We found that while models trained on videos of US children and models trained on Bangladeshi children both relied on many of the same clinically relevant features (eg, sensory seeking, stereotyped interests, and actions), some features were more prominent in one model compared to the other. For example, models trained on US data tended to rely more heavily on social participation and stereotyped speech, while models trained on Bangladeshi data relied more on eye contact. These patterns make sense, as raters could rely on a mutual understanding of the language (English) to evaluate behaviors like stereotyped speech and social interaction in US videos and may not have needed to rely as heavily on physical cues like eye contact, whereas when US raters viewed Bangladeshi videos, nonlanguage-based cues became more important. Even without the ability to confidently evaluate all aspects of the child’s behavior, the rater ensemble demonstrated that the set of behavioral features needed to make an accurate diagnosis of developmental delays, including ASD, may be narrower than previously thought. Nevertheless, the difficulty in assessing certain sociolinguistic patterns in the cross-cultural context may have been the cause of comparatively lower performance in the Bangladeshi dataset. We hypothesize that, when trained on annotations provided by raters who share a common linguistic and sociocultural background with the Bangladeshi children, our ensemble’s performance will improve and become comparable to the models trained and evaluated on the US dataset.

### Limitations

Although accuracy achieved using our source classifiers originally trained on US datasets was lower when applied to Bangladeshi videos, it still indicated a signal in the Bangladeshi dataset. The relatively low accuracy is most likely a result of three factors. First, these original classifiers were trained on clinical scoresheets, not on features obtained from live video data. Second, these scoresheets were obtained from formal clinical assessments of US children, and therefore they do not capture a culturally diverse set of behavioral nuances. Third, these classifiers were trained to distinguish between typically developing children and children with autism. However, this dataset consists of delays other than autism (eg, SLCs), which may be why these classifiers were unable to classify these cases with higher accuracy.

### Conclusions

Although the potential uses for a method of crowdsourced annotation and classification of developmental disorders like the one we established in this work are myriad, we wish to highlight a few uses. First, in areas where resources are scarce, and with a disorder like ASD, where early intervention is the key to successful treatment, our framework could be essential in performing cost-effective and reliable triage. Parents could send short home videos of their children to the cloud, at which point the video would be routed to several raters who perform feature tagging of the child’s behavior. Based on the raters’ previous annotation patterns and their associated models, the child would receive a predicted risk probability of developmental delay or ASD and a clinical team nearby could then be alerted, as appropriate. Since 2008, Dr Khan and her team have assisted the government to establish multidisciplinary Child Development Centers in tertiary hospitals across Bangladesh [26]. Fifteen such Child Development Centers are currently operational, whose chief mandate is to diagnose and provide appropriate management for a range of neurodevelopmental disorders including autism. However, in a country with population of 160 million, of whom an estimated 45% are within the pediatric age groups, access to reliable services can be limited. Formalization of the approaches documented here could enable broader reach and coverage through remote care while allowing resource-strapped clinical teams to deploy their efforts where they are needed the most.

An exciting second consequence of a deployment like this would be the steady development of a large corpus of annotated videos. No such dataset exists to date; however, the potential impact of such a dataset could be substantial. Modern algorithms from machine vision and speech recognition like convolutional and recurrent neural networks could use these annotations to *learn* features from the raw video and audio that are important for detecting developmental disorders, including ASD. Once trained, these models would dramatically accelerate the speed for detection of disorders and ability to accelerate the delivery of useful interventions.

Another important effect of such a pipeline would be that, with location-tagged videos, we could develop more accurate epidemiological statistics on the prevalence and onset of developmental disorders like ASD worldwide. Better information like this may increase awareness, positively impact policy change, and advance progress for addressing unmet needs of the children with developmental delays. This can have important applications in the developing world by helping countries identify the proportion of the population affected by such delays or impairments and therefore inform policy and gather actionable insights for health sector responses.

## Appendix

Multimedia Appendix 1 Formulae used in creating stacked rater-weighted classifiers.

## Abbreviations

ADI-R: Autism Diagnostic Interview-Revised
ADOS: Autism Diagnostic Observation Schedule
ASD: autism spectrum disorder
AUC: area under the curve
DSH: Dhaka Shishu Children’s Hospital
HIPAA: Health Insurance Portability and Accountability Act
MCHAT: Modified Checklist for Autism in Toddlers
SLC: speech and language condition
TD: neurotypical development
