# Supplementary material for: Detecting Developmental Delay and Autism Through Machine Learning Models Using Home Videos of Bangladeshi Children: Development and Validation Study
Source: J Med Internet Res. 2019 Apr 24;21(4):e13822. doi: 10.2196/13822 (PMC6505375; doi:10.2196/13822)

If we assume in the general case that our dataset consists of *K* raters, each of whom rated some subset (*N* videos) of the total videos of children, then given the vector of video annotations **x**^(i)^ for child *i*, rater *j*’s predictive model F_j_ would give a prediction


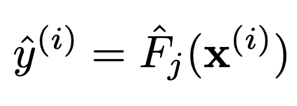


yielding a total accuracy of


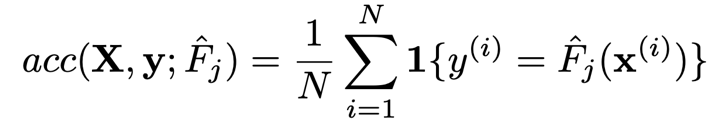


The training process would tune F_j_ to maximize expected accuracy on unseen data (which we estimate using 5-fold cross-validation):


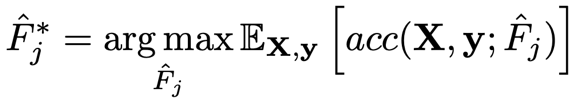


Meanwhile, the baseline classifier for rater *j* would naively classify each case as the majority class in the set and achieve an accuracy of


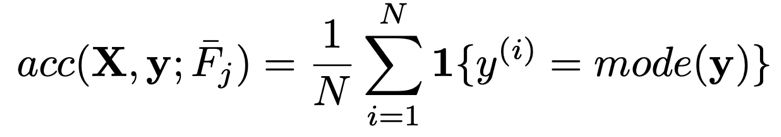


The gain in accuracy for rater *j*’s model would then be


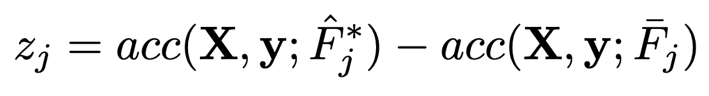


Then, after calculating z_j_ for each rater j = 1, 2, …, K, we would pass these values into the softmax function to generate rater-specific weights:


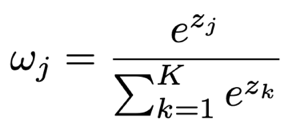


This ensures that all of the raters’ weights collectively sum to 1 so that the ensemble prediction will be a linear combination of each rater’s predictions. Using these weights, the final ensemble prediction for child *i* is given by the following equation:


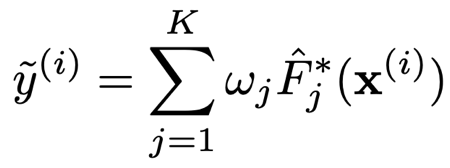

Supplement: Multimedia Appendix 1 [file jmir_v21i4e13822_app1.docx]
